# Tumeur stromale du mésentère: une cause inhabituelle d'une masse abdominale

**DOI:** 10.11604/pamj.2015.21.161.7181

**Published:** 2015-06-25

**Authors:** Mohamed Tarchouli, Ahmed Bounaim, Adil Boudhas, Moulay Brahim Ratbi, Bobby Nguele Ndjota, Abdelmounaim Ait Ali, Khalid Sair

**Affiliations:** 1 Service de Chirurgie Viscérale I, Hôpital Militaire d'Instruction Mohammed V, Université Mohammed V, Faculté de Médecine et de Pharmacie, Rabat, Maroc; 2 Service d'Anatomie Pathologique, Hôpital Militaire d'Instruction Mohammed V, Université Mohammed V, Faculté de Médecine et de Pharmacie, Rabat, Maroc

**Keywords:** Tumeur stromales gastrointestinales, mésentère, CD117, masse abdominale, Gastrointestinal stromal tumor, mesentery, CD117, abdominal mass

## Abstract

Les tumeurs stromales gastro-intestinales (GIST) sont les tumeurs mésenchymateuses les plus fréquentes du tractus digestif. Elles représentent une entité nosologique individualisée depuis la découverte de l'expression quasi-constante de la protéine c-Kit détectée par la coloration immunohistochimique de l'antigène CD117. Des tumeurs avec les mêmes caractéristiques morphologiques et immuno-phénotypiques peuvent rarement apparaître en dehors du tractus gastro-intestinal. Nous rapportons le cas d'une jeune patiente de 34 ans présentant une masse tumorale mésentérique se révélant être de nature stromale sans aucun contact avec la paroi intestinale. Il s'agit d'une localisation très rare des tumeurs stromales à laquelle il faut penser en préopératoire afin d'avoir une conduite thérapeutique adaptée et efficace.

## Introduction

Malgré qu'elles soient relativement rares, les tumeurs stromales gastro-intestinales sont les tumeurs mésenchymateuses les plus fréquentes du tractus digestif. Elles représentent une entité nosologique particulière caractérisée par l'expression quasi-constante à la surface des cellules tumorales d'un récepteur d'une tyrosine kinase communément appelé c-Kit ou CD117. Elles peuvent intéresser n'importe quel segment du tube digestif depuis l’œsophage jusqu’à l'anus mais siègent le plus souvent au niveau de l'estomac (60%) et l'intestin grêle (30%). Des tumeurs mésenchymateuses avec les mêmes caractéristiques histologiques et immuno-histochimiques peuvent apparaitre en dehors du tractus intestinal au niveau du mésentère, du grand épiploon ou du rétropéritoine. Elles sont dénommées tumeurs stromales extra-gastrointestinales(EGIST) représentant moins de 10% de l'ensemble des tumeurs stromales. Nous rapportons le cas rare d'une tumeur stromale du mésentère révélée par une masse abdominale en discutant, à travers une revue de littérature, les aspects cliniques et les stratégies thérapeutiques de cette pathologie assez particulière.

## Patient et observation

**Figure 1:**
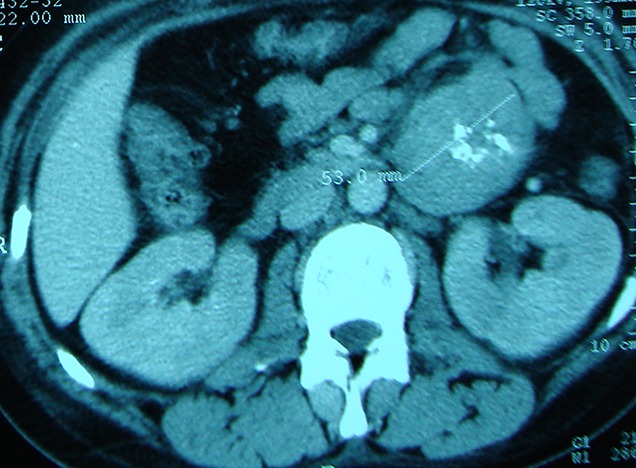
Coupe axiale de la TDM abdominale montrant une masse mésentérique indépendante des structures avoisinantes à contenu hétérogène renfermant quelques calcifications

**Figure 2:**
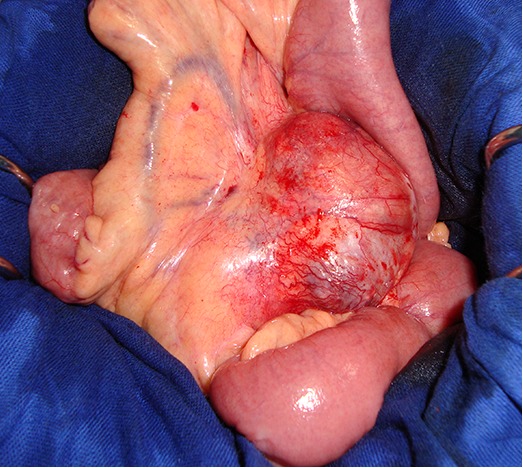
Vue opératoire montrant la tumeur localisée au niveau du mésentère sans contact avec la paroi intestinale

Nous rapportons le cas d'une jeune femme de 34 ans sans antécédents particuliers et jamais opéréeadmise pourdes douleurs épigastriques atypiques d'installation progressive évoluant depuis 6 mois. Ces douleurs étaient associées à des troubles intermittents du transit mais sans fièvre ni singes d'hémorragie digestive. Par ailleurs il n'y avait pas de notion de prise médicamenteuse ou d'altération de l’état général restant jusque-là conservé. L'examen clinique trouvait une sensibilité épigastrique et une masse palpable au niveau du flanc gauche. Cette masse était indolore, de consistance ferme, mobile par rapport au plan superficiel et fixe par rapport au plan profond. L’échographie abdominale avait objectivé une masse mésentérique faisant évoquer une adénopathie ou une tumeur bénigne de type schwanome. Une tomodensitométrie (TDM) abdominale a été ainsi réalisée pour mieux caractériser cette masse. Elle a objectivé une masse de consistance tissulaire à la racine du mésentère avec une prise de contraste partielle et un liseré de séparation des organes périphériques. Cette masse à contenu hétérogène comportant quelques calcifications refoule les anses intestinales et les vaisseaux mésentériques sans les envahir (Figure 1). En plus, il n'y avait pas de signes de nodules hépatiques ou d'adénopathies profondes. Les marqueurs tumoraux incluant l'alpha-fœto-protéine, l'antigène carcino-embryonnaire et le CA-19.9 étaient dans les limites normales et la fibroscopie œsogastroduodénale demandée dans le contexte d’épigstralgiesétait aussi sans anomalies. Une laparotomie, ainsi indiquée, a été réalisée sous anesthésie générale. L'exploration chirurgicale confirmait l'existence d'une masse d'allure tumorale de localisation mésentérique mais sans aucun contact avec les anses intestinales (Figure 2).

**Figure 3:**
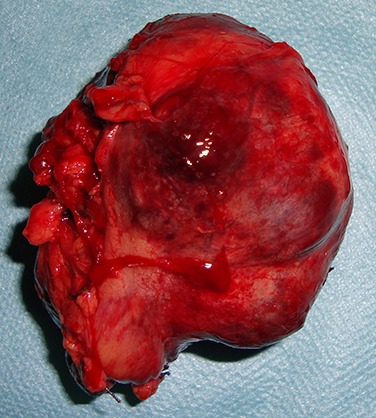
Pièce opératoire

**Figure 4:**
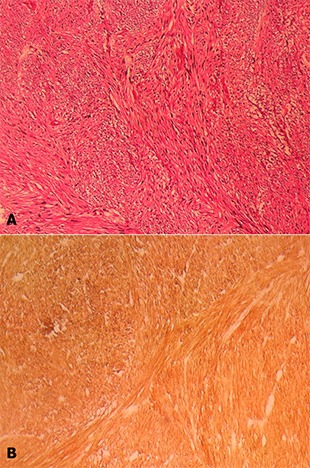
(A) étude histologique de la pièce opératoire montrant une prolifération tumorale faite de faisceaux anarchiquement enchevêtrés de cellules fusiformes (HE×40); (B) avec une expression diffuse du CD117 à l'immunohistochimie (x 20)

L'exploration du reste de la cavité abdominale n'a pas objectivé de lésions hépatiques ni de carcinose péritonéale. Le geste chirurgical consistait à une exérèse monobloc de la masse sans résection intestinale (Figure 3). La patiente a pu quitter l'hôpital 4 jours après l'intervention chirurgicale sans aucune complication post-opératoire. L’étude anatomopathologique de la pièce opératoire a mis en évidence une masse histologiquement solide mesurant 18x13cm avec une prolifération tumorale constituée de cellules fusiformes (Figure 4). Les limites d'exérèse étaient saines avec un index mitotique estimé à une mitose/50 champs au grossissement x40. A l’étude immunohistochimique les cellules exprimaient seulement l'anti CD117 (Figure 4). Alors que l'anti CD34, l'anti-AML, l'anti-P100, les anti-desmine et les anti-CK étaient tous négatifs. Le diagnostic d'une tumeur stromale du mésentère a été ainsi retenu et un traitement par imatinib a été instauré à raison de 400mg/j. Le contrôle régulier de la patiente avec un recul de six ans n'a pas montré de signes de récidive clinique ou radiologique.

## Discussion

Les tumeurs stromales gastrointestinales (GIST) sont des tumeurs mésenchymateuses rares qui expriment de façon quasi-constante à la surface des cellules tumorales le récepteur CD117 (protéine c-Kit) et qui sont actuellement clairement distinguées des autres tumeurs mésenchymateuses digestives n'exprimant pas ce récepteur telles les léiomyomes, léiomyosarcomes, neurofibromes ou schwannomes [1]. Les GIST sont présumées dériver des cellules interstitielles de Cajal. Ces cellules, décrites comme étant responsables du péristaltisme intestinal et qui expriment la protéine c-Kit à l’état normal, sont localisées dans la couche musculeuse du tractus digestif, souvent concentrées autour des plexus myentériques d'Auerbach [1]. Les tumeurs stromales extragastrointestinales(EGIST) se développent à partir des tissus mous intra-abdominaux et sont considérées primitives quand elles ne présentent aucune connexion avec la paroi du tractus gastro-intestinal [2]. Ces tumeurs représentent moins de 10% des tumeurs stromales et peuvent apparaitre au niveau du mésentère, du grand épiploon ou du rétropéritoine [3]. Leur histogenèse n'est pas encore totalement élucidée malgré qu'elles partagent les mêmes caractéristiques histologiques et immunohistochimiques avec leurs homologues gastrointestinales. L'expression de la protéine c-Kit par les EGIST suggère la présence de la cellule interstitielle de Cajal en dehors du tractus gastro-intestinal ou plutôt la capacité des cellules mésenchymateuses d'exprimer ce même phénotype d'une façon aberrante [2, 4]. Miettinen et al pensent que les EGIST épiploïques et mésentériques proviendraient respectivement de l'estomac et l'intestin grêle et qui se détachent, pour une raison inconnue, de la paroi gastro-intestinale au cours de leur développement [3]. Mais récemment, certains auteurs ont montré que ces tumeurs peuvent provenir de cellules souches mésenchymateuses multipotentes avec une expression de la protéine c-Kit et un aspect histologique similaire à celui des cellules interstitielles de Cajal conduisant à considérer les EGIST comme une entité analogue aux GIST [5, 6]. Vu leur localisation profonde et le développement extraluminal, les tumeurs stromales mésentériques restent longtemps silencieuses. Toutefois, des douleurs abdominales vagues et/ou la palpation d'une masse abdominale peuvent être des signes d'appel comme c'est le cas dans notre observation. L'imagerie médicale (TDM + IRM) apporte une contribution indéniable dans le diagnostic préopératoire des EGIST en permettant, outre la visualisation de la tumeur, de guider une ponction biopsie à l'aiguille fine à visée diagnostique. Selon Ortiz-Rey et al. [7], ce geste relativement simple et bénéfique est d'indication courante dans les EGIST. Chez notre patiente, la TDM a objectivé une masse à la racine du mésentère, cependant aucune biopsie n'a été effectuée afin d’éviter le risque d'une éventuelle dissémination tumorale dans la cavité abdominale. Selon les données de la littérature, différents paramètres ont été proposés pour prédire le potentiel malin des EGIST, tels que la taille de la tumeur, la cellularité et l'activité mitotique.

Le système de gradinghistopronostique utilisé pour les GIST, combinant index mitotique et taille tumorale [8] n'est pas applicable pour les EGIST qui sont le plus souvent de grande taille (≥ 10 cm) au moment de leur découverte [9]. La localisation de la tumeur semble être un facteur pronostique indépendant sachant que les tumeurs stromalesépiploïques sont réputées plus agressives que celles mésentériques [3, 9]. Alors que dans une étude récente, Yamamoto et al. [6] définissent trois grades pronostiques sur la base de l'index mitotique et de l'indice de prolifération tumorale Ki67: un groupe de haut risque de malignité associant un index mitotique ≥5/50 champs au fort grossissement (CFG) et un Ki-67 ≥10%, un groupe de risque de malignité intermédiaire avec un index mitotique ≥5/50 CFG et un Ki-67< 10% ou un index mitotique <5/50 CFG et un Ki-67 ≥ 10%, et un groupe de faible risque de malignité associant un index mitotique <5/50 CFG et un Ki-67 < 10%. Plus récemment, quelques auteurs ont suggéré d'inclure le profil moléculaire de la tumeur comme un facteur pronostique validé des EGIST [10]. L'exérèse chirurgicale complète de la tumeur avec des marges saines reste le traitement de première intention des EGIST non métastatiques. Durant l'intervention chirurgicale, il est important de veiller à réaliser une résection monobloc de la masse tout en vérifiant l'absence de la moindre adhésion à la paroi gastrointestinale. Ainsi l’évolution dépend non seulement du grade histopronostique mais aussi de la qualité du geste chirurgical initial. Par ailleurs, l'utilisation de l'imatinibmesylate, un inhibiteur des tyrosines kinase, a révolutionné la prise en charge des GIST en particulier en situation de récidive ou de métastases. En effet, l'expression de la protéine c-Kit et les mutations du gène c-Kit, semblables à celles retrouvées au niveau des GIST, impliquent la possibilité d'utiliser cette molécule dans le traitement des EGIST avancées ou métastatiques [6]. Actuellement l'imatinib est également recommandé comme traitement adjuvant après une chirurgie même complète d'une tumeur stromale gastrointestinale de haut risque de malignité afin de prévenir les récidives. Ces recommandations sont généralement applicables pour les EGIST car une étude spécifique dans ce groupe de malades n'est pas réalisable compte tenu de la rareté de cette maladie. Chez notre patiente, malgré une résection chirurgicale complète, un traitement adjuvant par l'imatinib a été instauré vu la taille et la localisation de la masse jugés prédictifs d'un haut risque de malignité.

## Conclusion

Les EGIST sont des tumeurs mésenchymateuses rares ayant le même profil morphologique et phénotypique que celui des GIST. Longtemps silencieuses, elles sont le plus souvent découvertes après avoir atteint une grande taille. La résection chirurgicale complète reste le traitement de choix malgré des avancées significatives de la thérapie ciblée. Leur potentiel évolutif et ainsi les modalités thérapeutiques sont moins bien codifiés et suivent en général les recommandations appliquées dans la prise en charge de leurs homologues digestifs. Par ailleurs d'autres études sont nécessaires pour une compréhension plus profonde de cette pathologie.
